# The childbirth experience: obstetric and psychological predictors in Italian primiparous women

**DOI:** 10.1186/s12884-019-2561-7

**Published:** 2019-11-15

**Authors:** Valentina Fenaroli, Sara Molgora, Serena Dodaro, Alessandro Svelato, Livia Gesi, Giulia Molidoro, Emanuela Saita, Antonio Ragusa

**Affiliations:** 1 Department of Psychology, Università Cattolica del Cacro Cuore, Largo Gemelli 1, 20123 Milan, Italy; 2 Department of Obstetrics and Gynecology, San Giovanni Calibita Fatebenefratelli Hospital, Tiber Island, Rome, Italy

## Abstract

**Background:**

The experience of childbirth crucially impacts a mother’s psychological well-being and the mother-infant relationship. It is recognised that negative births can be linked to different forms of discomfort, both for the mother as well as for the infant. This prospective longitudinal study aimed to study the effect of obstetric and psychological variables on women’s subjective experience of childbirth.

**Methods:**

111 primiparous Italian women completed a set of questionnaires at 38–40 weeks of pregnancy (Time 1) and 1–5 days after childbirth (Time 2). Sociodemographic and obstetric information were collected. Data about the childbirth were obtained from the mother’s ward birth records. Women completed the Wijma Delivery Expectancy/Experience Questionnaire both before and after childbirth.

**Results:**

The subjective experience of birth was significantly predicted by the duration of the expulsive phase (β = .26; *p* < .05), the use of epidural analgesia (β = .21;  *p*< .05) and by fear of birth (β = .21; *p* < .05). The effect of mode of birth and duration of the dilatation phase on women’s birth experience was not found.

**Conclusions:**

In our study, neither instrumental childbirth nor caesarean section have a significant effect on women’s birth experience. Instead, both a longer expulsion phase and epidural analgesia contribute to the negative experience. Moreover, the higher the fear of birth, the worse the women’s emotional experience. These findings confirmed the role of obstetric and psychological variables on birth experience. More investigation about this topic could be useful to develop specific interventions to prepare women for birth.

## Background

Childbirth is a crucial event in a woman’s life. The woman’s emotional and cognitive experience of birth is recognized as having a significant impact on her postpartum physical and psychological state [1] and on her first interactions with the infant [2]. A positive childbirth experience can improve maternal well-being and facilitate mother-infant bonding, while a negative experience can lead to a condition of psychological distress as well as to severe forms of disease such as postpartum depression [3] or post-traumatic stress symptoms [4], often connected with the level of pain perceived during childbirth [5]. These conditions have inevitable long-term consequences on the relationship with the child in addition to its well-being and development [6].

Existing literature has investigated variables associated with the birth experience, focusing on both obstetric-related variables [7, 8] and psychological variables related mainly to expectations about childbirth [8, 9]. As regards obstetric variables, mode of birth – specifically an emergency caesarean section (CS) or an instrumental vaginal birth – has been found to have a negative impact on women’s birth perceptions [9–13]; however, some studies did not confirm this relationship [14], and the topic is still debated.

Labor induction and labor duration [8, 15] have been linked to low maternal satisfaction with birth, while pain relief seems to enhance the maternal experience [16]. Even in this case, however, results are still divergent, in that some studies have shown that women who are most satisfied with the birth experience have had a natural birth, without pharmacological pain control [8, 14, 17, 18].

As regards the impact of psychological variables on the birth experience, literature has largely focused on the role that expectations could play in shaping the subjective emotional experience. Some studies have found that positive expectations predict higher childbirth satisfaction, and negative expectations predict worse emotional experience [8, 14, 19]. However, also in this case it should be emphasized that results are still contradictory, and some research did not find this relationship [20]. Some authors argue that unfulfilled expectations about birth may affect the experience, generating disappointment and adverse emotional outcomes [21]. Among childbirth expectations, fear of childbirth (FOC) has been shown to be a predictive variable for the fear experienced during birth and negative birth experience [22, 23]. FOC plays an important role not only in the mother’s emotional experience, but also in the development of both the child and its relationship with the mother [24]. A study conducted by Elvander et al. [19] revealed that women with high levels of FOC who give birth by unplanned CS have a 12-fold increased risk of reporting a negative birth experience. Results, however, are still inconsistent, revealing the need for additional focus on the subject [21].

The aim of the present study was to analyze the effect of obstetric-related childbirth variables (i.e., mode of birth, duration of dilatation and expulsive phase, labor induction, use of epidural analgesia) and FOC on women’s subjective experience of childbirth.

## Method

### Procedure and participants

Italian nulliparous women at the end of pregnancy were eligible for this prospective longitudinal study. Other inclusion criteria were: being over 18 years old and fluent in Italian, with a singleton pregnancy, and a planned normal vaginal birth. Participants were informed about the overall research aims and methodology. A midwife distributed a questionnaire to pregnant women who agreed to participate and provide signed written consent before they attended a routine examination, scheduled with the Italian National Health Service, at 38–40 weeks of pregnancy. The questionnaire was filled out on site (*Time 1)*. Within 1–5 days after childbirth (*Time 2*), a midwife distributed to women who had participated in *Time 1* a second questionnaire during the post-partum recovery; in case a woman had already been discharged, the midwife called her, and the questionnaire was filled out directly over the telephone. The study was granted ethical approval by the Institution’s Ethics Review Board (2013). Obstetric data on labor and childbirth were obtained from the maternity ward records.

Recruitment of participants took place between January, 2016 and November, 2016 at the Department of Obstetrics and Gynecology of the General Hospital of Massa Carrara in Italy, which is an urban community hospital serving a multi-ethnic patient population under the auspices of the National Health Service (1500 deliveries/year). In Italy maternity and childbirth assistance is completely public. A public hospital does not charge anyone for giving birth or for any emergency procedures that may occur during labor and delivery. Almost all childbirths in Italy take place in public or private hospitals; giving birth at home is a practice that is increasing in frequency but is still rare.

217 women who met the inclusion criteria were recruited, and 211 filled out the questionnaire at *Time 1*. Among these, 117 women filled out the questionnaire at *Time 2*. Six women were excluded because their questionnaires at *Time 2* were incomplete*.* The final sample was thus composed of 111 women with both *Time 1* and *Time 2* questionnaires completed. Women who did not take part in *Time 2* did not differ significantly from women with *Time 1* and *2* in terms of age, socioeconomic status, and pregnancy progression. No significant difference was found even with respect to fear of childbirth. However, women who declined to fill out the questionnaire at *Time 2* after childbirth had significantly fewer natural vaginal births and more urgency/emergency CS and births at moderate risk; in addition, the dilatation phase for these women was longer than for women with *Time 1* and *2* (see Table 1)*.*

**Table 1:** Medical-obstetric informations

	%
Prior to pregnancy	
Obstetric problems prior to pregnancy (fibromas, endometriosis, polycystic ovary syndrome, etc.)	10.7
Two or more previous miscarriages	21.6
Pregnancy	
Severe complications during pregnancy (preeclampsia, hypertension, diabetes, etc.)	10.4
Moderate complications during pregnancy (bleeding in third trimester, placental abnormalities, infections, etc.),	49.3
No complication during pregnancy	40.3
Childbirth	
Spontaneous vaginal delivery	66.4
Operative vaginal delivery	14.2
Urgency/emergency cesarean section (CS)	19.4
Childbirth at moderate risk (i.e., premature rupture of membranes, oxytocin, abnormal fetal presentation)	35.1
Induction (> 41 + 5th week)	7.1
Epidural analgesia	45.4

### Measures

At *Time 1*, women completed:

- a data sheet describing age and socioeconomic status, measured by the Hollingshead Index of Social Position [25];

- the *Wijma Delivery Expectancy Questionnaire* – WDEQ(A) – [26], the most frequently used self-report measurement of childbirth expectations, with particular attention to fear of birth. The original instrument consists of 33 items using Likert scale ratings from “not at all” (score 0) to “extremely” (score 5). Following a validation study conducted with a large sample of 522 Italian primiparous women [27], the reduced 14-item version of the WDEQ(A) was found to work best on an Italian population: therefore, this version was administered in the present study. For the 14-item version, the total score ranged between 0 and 70, with higher scores indicating greater fear of childbirth. The WDEQ(A) showed good reliability (Cronbach alpha = .81).

- a data sheet describing previous miscarriages and pregnancy progression.

Labor and childbirth information was then obtained from the maternal birth records. These data included: [1] duration of dilatation phase (i.e., first stage of labor), defined as the beginning of frequent contractions (≥3 contractions per 10 min and a cervix dilation of approximately 4 cm) [2]; duration of the expulsive phase (i.e., second stage of labor) [3]; induction of labor (for pregnancies beyond the 41 + 5th week), which included amniotomy, endocervical placement of a catheter, and oxytocin or prostaglandin administration (selected on the basis of the clinical evaluation of the woman) [4]; use of epidural analgesia during labor (started at any time during labor); and [5] mode of childbirth: spontaneous vaginal childbirth, instrumental vaginal childbirth (with vacuum assistance), or emergency caesarean section (CS).

At *Time 2* women filled out:

- the *Wijma Delivery Experience Questionnaire* – WDEQ(B) [26], measuring the childbirth experience as perceived by the women; the higher the score, the worse the childbirth experience and fear felt. The WDEQ(A) and WDEQ(B) have the same items, referring to upcoming labor and birth for version A and the experienced labor and birth for version B. As regards the WDEQ(A), the 14-item version [27] was used, showing good reliability (Cronbach’s alpha = .84).

### Data analysis

Data were analyzed using SPSS version 21. Continuous variables were expressed as mean ± standard deviation and categorical variables as percentages. The normality of distributions of WDEQ(A) and WDEQ(B) scores were verified. The difference between the WDEQ(A) and WDEQ(B) mean scores was tested through a paired sample T-test.

The effect of FOC and obstetric-related childbirth variables (i.e., mode of birth, duration of dilatation and expulsive phase, labor induction, epidural analgesia) on subjective experience of childbirth was tested through a linear multiple regression model. Possible multicollinearity was tested by calculating the variance inflation factor (VIF) and tolerance values.

When predictors were dichotomous (mode of birth, labor induction, epidural analgesia), they were recoded as dummy variables [28]. With reference to the mode of birth, instrumental vaginal childbirth and emergency CS were merged into a single category. Although they present significant differences from a medical-obstetric point of view, some studies point to psychological similarities that make it possible to consider these two experiences as comparable in regard to the women’s feelings [29].

## Results

Women had an average age of 32.29 years (SD = 5.23, range = 19–46); the average Hollingshead occupation score was 4 (*SD* = 2.8) corresponding to technician, semi-professional, or small business owner, with a range from 0 (housewife, student, unemployed) to 9 (doctor, lawyer, manager, etc.); 66.4% of women had a spontaneous vaginal birth; 14.2% gave birth vaginally with vacuum assistance; 19.4% had an urgency/emergency caesarean section (CS); 64.9% of women gave birth without complications, 35.1% with moderate risk factors (i.e., premature rupture of membranes, oxytocin, abnormal fetal presentation, etc.); finally, 45.4% of parturient women received epidural analgesia. Other medical-obstetric variables of the sample are shown in Table 1.

Considering the average values of WDEQ(A) and WDEQ(B), WDEQ(B) scores were significantly lower than the WDEQ(A) scores (t = 2.37; df = 110; *p* < .05), as shown in Table 2. No significant correlations were found between demographic variables (i.e., SES and age) and FOC, nor between demographic variables and experience of childbirth.

**Table 2:** WDEQA e WDEQB: Descriptive data

	Mean	SD	Skewness		Kurtosis	
			Statistic	S. E.	Statistic	S. E.
WDEQ(A)	26.33	9.35	.20	.17	.41	.33
WDEQ(B)	23.46	11.69	.50	.22	.01	.44

The multiple regression model showed that WDEQ(B) was significantly predicted by epidural analgesia, the duration of the expulsive phase of labor, and WDEQ(A) (Table 3). For all predictors, the tolerance values were between .65 and .97, the VIF between 1.03 and 1.60; no multicolliearity problem emerged. The model (F _(6, 98)_ = 4.72; *p* < .001) explains 23% of the total variance of the dependent variable (R^2^ = .23). Conversely, mode of birth, labor induction, and duration of the dilatation phase did not significantly predict quality of the women’s subjective experience.

**Table 3:** Regression coefficients

Model	Unstandardized Coefficients		Standardized Coefficients	t	Sig.
	B	Std. Error	Beta		
(Constant)	10.73	3.75		3.11	.001
Type of delivery	2.84	3.56	.09	.80	.43
Dilatation (min)	−.00	.01	−.04	−.42	.67
Expulsive phase (min)	.06	.03	.23	2.06	.04
Epidural analgesia	5.02	2.35	.21	2.13	.03
WDEQA	.26	.11	.21	2.34	.02

a. Dependent Variable: WDEQB

## Discussion

Similarly to previous research, our results confirm that a woman’s subjective experience of birth depends on both obstetric and psychological variables. While according to some studies, many of which were conducted in the Scandinavian and Australian context [30, 31], CS or instrumental births contribute to making the childbirth experience more stressful [9] – with more negative thoughts and emotions, and with long-lasting effects on women’s self-efficacy and self-control [32] – in our research, mode of birth did not significantly shape the woman’s subjective experience. Specifically, women with vaginal birth did not have a more positive emotional and cognitive experience than women who had a vacuum-assisted birth or who had to undergo unexpected surgical interventions. To explain this inconsistency, we can speculate that the relationship between mode of birth and birth experience is not direct, but, rather, it is mediated by psychological variables, both individual and context-related. For example, a woman could negatively perceive an urgency/emergency CS or an instrumental childbirth because she feels ineffective, or because she had a different birth experience than expected. At the same time, however, it is possible that women who gave birth with CS or with vacuum assistance felt well supported by the health practitioners and, for this reason, their overall experience can be positive. This topic needs further investigation.

What adversely affects the birth experience in our study is the duration of the expulsive phase of birth. If the first stage of labor does not significantly affect women’s emotional and cognitive appraisal of childbirth, the longer the second stage, the worse the woman’s subjective experience. In other words, it seems that it is the final stage of the whole labor process that weighs heavily on the quality of the woman’s emotional experience. Some psychological research on memory processes may offer a contribution to explaining this result. For example, some studies on memories of unpleasant medical procedures [33] suggest that patients’ memories of painful medical procedures reflect the intensity of the final part of the experience more than the whole experience in itself. In a similar way, it is possible that the pain experience during the second stage of labor has a deeper effect on women’s memories and emotions than the first stage, even if the latter lasts longer. We can also hypothesize that one-to-one assistance provided by the midwife to the woman during the first stage of labor – which in nulliparous women could last several hours – contributes to reducing the impact of pain on the woman’s birth satisfaction. Indeed, literature underscores that relational variables, such as practical and emotional support from a partner or a midwife during labor and birth, can influence the childbirth experience as well [34, 35]. In this way, the support from hospital staff may ultimately help mothers-to-be feel more self-confident and able to cope with the labor process [36, 37].

The use of epidural analgesia was also implicated in women’s perception of their birth experience, but in a counterintuitive direction. A number of studies have found that less pain experienced during labor and childbirth is related to more satisfaction [38]. For the women in our study, use of pharmacological pain relief fostered birth experiences that were less positive as compared to those in which the woman had a natural childbirth without pain relief. Other studies have analyzed the complex relations between childbirth satisfaction, labor pain, and analgesia, confirming that satisfaction during labor is not necessarily directly correlated with pain or pain relief [31, 39, 40]. It is hypothesized, for example, that some women require analgesia because of a prolonged and difficult labor, so they may appraise their birth as stressful or less satisfactory than women who give birth naturally. Pain relief, therefore, is one, but not the most significant, factor that contributes to the evaluation of the birthing experience. In the same way, women who have vaginal natural births may perceive more personal efficacy in the birth process and more ability to give birth even when facing difficulties. Van der Gucht and Kiara [41] highlight how some parturient women view the pain of giving birth as a challenging life element, able to enhance their coping ability. Some sociocultural elements can explain these results as well [42]. In Italian society, especially in past decades, for example, the woman’s ability to tolerate birth pain was often embraced as part of a “full childbirth experience” and a sign of a woman’s self-efficacy. Some women could, therefore, feel efficacious in knowing that they have not “surrendered” to the relief of pain, which results in a more positive birth [43].

Our results show the emergent importance of expectations of childbirth as a predictor of birth experience: the higher the fear, the worse the women’s emotional and cognitive experience. These results are consistent with other studies that highlight how thoughts and feelings about childbirth affect how a woman evaluates the experience and how she copes with it [14, 19]. This result is more important if we consider its practical implications: antenatal classes should help women feel powerful and able to cope with labor and birth, allowing them to develop more positive expectations; in this way, the childbirth experience could probably become more positive for parturient women.

Certainly, the predictors that emerged in our study explain only a limited part of the global birth experience (23% of total variance) and fail to explain the variables that affect women’s thoughts and emotions. The childbirth experience is a multidimensional construct, which requires the consideration of many variables. For example, several studies show how attentive assistance from midwives has a pivotal role in promoting positive birth experience [36], helping the women feel safe and cared for [44]. Moreover, some literature highlights how individual variables, such as sense of control or self-efficacy, could affect coping strategies during labor and subsequently influence birth satisfaction [44]. The role of relational and individual psychological variables in the childbirth experience should be further studied.

Our study has some limitations. The first one is certainly represented by the loss at *Time 2* of more than half of the sample. One aspect that must be highlighted is that these women had more deliveries at moderate risk and more medical-obstetric complications (i.e., urgency/emergency CS) than the women with *Time1* and *Time 2*. Thus, it is possible that women with a negative experience withdrew from the study because of their critical physical or psychological condition. This is a frequent bias in clinical studies which risks weakening the results obtained, limiting their generalizability. For these reasons, we think that our results should be interpreted with caution but that they offer some preliminary interesting starting points for future investigations in which a way must be found to avoid/bypass this methodological bias. Further research will allow us to test, for example, whether even in the case of at-high-risk births, with obstetric/medical complications, women’s subjective experience is affected by the variables that emerged in our study.

In addition, the obstetric characteristics of our sample are representative of the Tuscan population, a region in central Italy, but not of the overall Italian population. Tuscany, in fact, has a CS rate of 21.7%, which is lower than the national rate, which is around 35% [45]. We should clarify that health care in Italy is managed at a regional level with strongly independent policies, and childbirth statistics vary greatly from one region to another. For this reason, it would be useful to expand the research to different Italian regions so that the results could be made generalizable to the entire Italian population. Moreover, as already described, this study did not consider the impact of intra-partum variables, for example, the impact that relational and organizational elements may have on a woman’s subjective birth experience [46]. Furthermore, the neonatal outcome could also have an impact on women’s emotional birth experience. Some risk factors for infant health (for example, gestational age, a low PH index, etc.) might worry or frighten the woman and could change her emotional experience. It is important to take this issue into account for future studies. Finally, our sample is rather small, and we do not have the exact value of the response rate. Although the research was carefully explained to all the women who met the inclusion criteria, the response rate should prompt us to reflect on factors that may have interfered with recruitment.

## Conclusion

Despite the limitations described above, our study confirms the role played by obstetric variables in shaping the woman’s subjective experience of childbirth, although in a partially unexpected direction. In particular, it is not the type of birth – neither a CS nor an operative birth – that affects the subjective birth experience, but the duration of the expulsion phase and the use of analgesia. The woman’s experience is more positive when the expulsion phase lasts less time and when analgesics are not used. Our results also demonstrate that psychological elements, linked to expectations about childbirth and, more specifically, to the level of fear with which women approach the event, are also important. Future insights into this aspect should better explain the impact of fear. Our results offer an opportunity to look specifically at the Italian context, which can lead to comparisons with what has been developed in other countries; in particular, we refer to specific interventions that include the direct involvement of midwives in preparing pregnant women for birth [47].

## Data Availability

The datasets used and/or analyzed during the current study are available from the corresponding author upon request.

## Abbreviations

CS: Cesarean section
FOC: Fear of childbirth
WDEQ(A): Wijma Delivery Expectancy Questionnaire
WDEQ(B): Wijma Delivery Experience Questionnaire
